# Harnessing Pharmacokinetic Modeling to Develop a Long-Acting Subcutaneous HIV Treatment Platform for Young Children

**DOI:** 10.3390/pharmaceutics18050522

**Published:** 2026-04-24

**Authors:** Daniel Oliveira, Daniela Cruz, Leanna P. K. Levin, Linying Li, Chasity A. Norton, Georgina Dobek, Xiaolei Wang, Ronald Veazey, Meagan Watkins, Amanda P. Schauer, Julie B. Dumond, Leah M. Johnson, Mackenzie L. Cottrell

**Affiliations:** 1 Department of Pharmacotherapy and Experimental Therapeutics, University of North Carolina Chapel Hill, 120 Mason Farm Road, Genetic Medicine Building 7361, Chapel Hill, NC 27599, USA; 2 RTI International, 3040 E Cornwallis Rd., Research Triangle Park, NC 27709, USA; 3 Department of Comparative Medicine, Tulane University School of Medicine, 1430 Tulane Avenue, New Orleans, LA 70112, USA; 4 Tulane National Biomedical Research Center, Division of Comparative Pathology, 18703 Three Rivers Road, Covington, LA 70433, USA; 5 Department of Pharmacy: Clinical and Administrative Sciences, University of Oklahoma, 1110 N. Stonewall Ave., CPB 250, Oklahoma City, OK 73117, USA

**Keywords:** pharmacokinetics, long-acting, pediatric, antiretroviral, HIV treatment, deconvolution

## Abstract

**Background:** Long-acting drug delivery strategies could augment pediatric human immunodeficiency virus (HIV) treatment effectiveness by bypassing population-specific challenges such as adherence. We harnessed pharmacokinetic (PK) modeling to develop a biodegradable, subcutaneous (SQ), reservoir-style implant for HIV treatment in 2–5-year-old children. **Methods:** Plasma was collected from New Zealand White rabbits over 30 h after a single intravenous (IV) bolus of bictegravir (BIC, 0.75 mg/kg), islatravir (ISL, 5 mg/kg) and/or emtricitabine (FTC, 30 mg/kg) then over a year after subcutaneous insertion of two to three implants eluting these antiretrovirals. Plasma antiretrovirals were quantified by HPLC-MS/MS and population PK models were fit to the IV PK profile to derive a mean unit impulse response (UIR). UIR was used to numerically deconvolve SQ absorption rate from the implant PK profile. SQ dosing rates were translated to pediatric plasma concentrations using published clinical PK parameters. **Results:** BIC, FTC, and ISL PK profiles were best described by two-compartment models. Each implant achieved quantifiable plasma concentrations for >360 days. Median SQ absorption rates (μg/day) at 3, 6 and 12 months of implantation were 116, 98 and 71 for BIC; 116, 37 and 5 for ISL; and 236, 116 and 24 for FTC. These 6-month dosing rates translated to pediatric plasma concentrations of 24 ng/mL BIC, 0.14 ng/mL ISL, and 0.7 ng/mL FTC. **Conclusions:** Our novel long-acting delivery platform exhibited antiretroviral SQ dosing rates for ≥6 months that are anticipated to achieve plasma concentrations in children within an efficacious range warranting further development for pediatric HIV treatment.

## 1. Introduction

Of the approximately 1.38 million children aged 0–14 living with HIV globally, only 55% were receiving life-saving antiretroviral therapy (ART) in 2024 [1]. Compared with adults, pediatric patients often experience suboptimal health outcomes on standard HIV treatment regimens, with early childhood (particularly 0–4 years of age) associated with higher mortality from AIDS-related complications [2]. The Department of Health and Human Services (DHHS) treatment guidelines recommend daily oral treatment for antiretroviral-naïve children aged 2–5 years, using an initial regimen that combines two nucleoside reverse transcriptase inhibitors (NRTIs) with an integrase strand inhibitor (INSTI) [3]. However, adherence to this daily oral treatment may be uniquely challenged among young children due to difficulties swallowing pills and/or the poor palatability of oral suspensions [2].

Long-acting antiretrovirals (LA-ARTs) that facilitate extended dosing intervals beyond daily administration have received FDA approval for HIV treatment in adults. To date, Cabenuva (cabotegravir/rilpivirine) is the only LA injectable regimen for full HIV treatment approved by the FDA [4]. However, the safety and efficacy of Cabenuva is only now being evaluated in pediatric patients in the Phase I/II IMPAACT 2036/CRAYON trial [5]. Despite potential advantages for children, these regimens still present difficulties to implement, especially within resource-limited settings, given the requirements for large-volume intragluteal injections administered within a healthcare setting every 1–2 months, dependence on cold-chain storage, and limited reversibility if an adverse drug event occurs. Additionally, Cabenuva exhibits a first-order absorption profile, requiring excessive front-loaded drug exposure to ensure therapeutic concentrations are maintained throughout the dosing interval. Finally, the lengthy pharmacokinetic (PK) tail of Cabenuva could lead to antiviral resistance in patients who fall out of care or delay injections [6].

LA delivery of antiretrovirals (ARVs) via a subcutaneously (SQ) administered drug-eluting implant aligns well with certain preferred user characteristics for pediatric HIV treatment valued by health care providers and caregivers of children living with HIV [7,8]. We have previously demonstrated the utility of a reservoir-style, membrane-controlled, biodegradable implant comprising poly-(ε-caprolactone) (PCL), an aliphatic polymer already used in other approved drug and biomedical products, to deliver ISL for >6 months both in vitro and in vivo (rats) through a zero-order absorption profile [9,10]. Although originally designed for HIV pre-exposure prophylaxis in adults, this implant platform is well-suited for therapeutic applications, since the implant can hold drug quantities that exceed solubility constraints, thereby maintaining a saturated reservoir that enables zero-order drug release. Upon fluid permeation through the polymer and into the reservoir, the active pharmaceutical ingredient (API) dissolves to saturation, after which the dissolved API diffuses across the PCL. Furthermore, drug release is independent of polymer degradation, as the bulk-mode hydrolysis of PCL occurs over extended timescales (e.g., years) before the loss of mechanical integrity.

Scaling the dose of LA-ART products to ensure therapeutic plasma ARV concentrations in the target population is a critical consideration in product development. Numerical deconvolution is a well-established mathematical tool that can estimate the absorption kinetics of LA formulations by coupling drug clearance information with observed PK profiles. This technique has been approved for use in drug development by the FDA for over two decades and serves as the foundation for in vitro to in vivo correlation (IVIVC) modeling, which is one of the original new approach methodologies (NAMs) used to replace traditional animal studies. Indeed, in 1997 the FDA published specific guidance outlining how IVIVC can be used to establish bioequivalence and support many types of drug applications such as New Drug Applications (NDAs), Abbreviated New Drug Applications (ANDAs), and Antibiotic Drug Applications (AADAs) [11].

**Figure 1:**
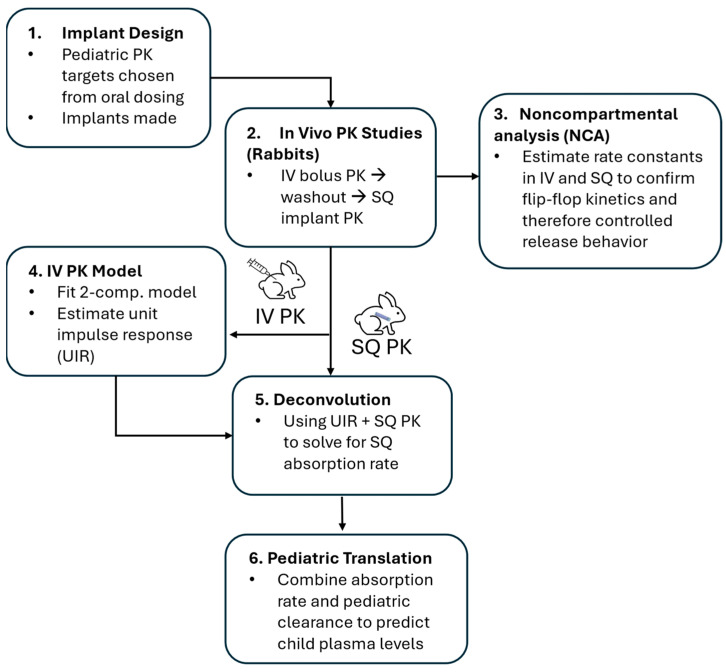
Methodological schema for preclinical PK modeling study.

Building upon our previous findings in rats, the purpose of this research is to extend the potential clinical application of our reservoir-style implant for an HIV treatment regimen by assessing the PK of a complete three-drug HIV treatment delivered via intravenous (IV) bolus then SQ implant. Here we employ animal and in silico models to guide formulation development and translate the resulting implant dosing to clinical PK within our target pediatric population (Figure 1). Rabbit was pragmatically selected as the largest of the commonly employed small animal models in drug development since their larger size facilitated the placement of multiple implants between scapulae on the dorsal side.

## 2. Methods

Materials: Emtricitabine (FTC), Bictegravir (BIC), and Islatravir (ISL) were purchased from Boc Sciences (Shirley, NY, USA), AstaTech Inc. (Bristol, PA, USA), and Wuxi AppTec (Wuhan, China), respectively. Excipients Super Refined^TM^ Castor Oil (Cat# SR40890) and PEG_40_ Castor Oil (Castor Oil Etocas 40-SS-(MH), Cat# ET48333) were purchased from Croda (Snaith, UK) and Glycerol (Cat#G6279) was purchased from Sigma Aldrich (St. Louis, MO, USA).

Implant Fabrication: Medical-grade PCL pellets (PURASORB PC-17) were procured from Corbion (Amsterdam, The Netherlands), with a manufacturer-reported intrinsic viscosity between 1.5 and 1.9. Our in-house measurements of PURASORB PC-17 had previously shown a weight-average molecular weight (Mw) of 106 kDa and a polydispersity (PD) of 2.6, as determined by gel permeation chromatography [12]. PCL tubes were fabricated via a hot-melt, single screw extrusion process using solid PCL pellets at GenX Medical (Chattanooga, TN, USA). Before the extrusion process, all the PCL pellets were dried in a compressed air dryer at 60 °C for 4 h. All tubes measured 2.5 mm in outer diameter (OD), as determined by using a 3-axis laser measurement system and light microscopy at GenX Medical. In previous work, we have demonstrated that extruded PC-17 tubes exhibited flexible and mechanically robust behavior, with an ultimate tensile strength of 41.5 ± 2.6 MPa [12]. All implants were fabricated in a biosafety cabinet under aseptic conditions using a previously reported method [12]. In summary, PCL tubes were first marked and trimmed to the correct length to achieve an implant with the desired reservoir (25 mm or 40 mm) with 3 mm of headspace at both ends for sealing. A heat seal was applied to one end of the empty PCL tubes to establish an initial injection seal, resulting in a cylindrical PCL closure approximately 2 mm in length. The tubes were weighed prior to the filling process. Drug formulations were prepared by mixing excipients with drugs, grinding them to a uniform paste, and loading them into the tubes with a syringe (ISL and FTC) or rod (BIC). After filling, residual paste was cleaned from the headspace, and the tubes were weighed again before final sealing. The ISL-containing implants comprised 40 mm length, 200 µm wall thickness, and a drug formulation of 1:1 weight ratio of ISL to PEG_40_ castor oil excipient. The FTC-containing implants comprised 40 mm length, 300 µm wall thickness, and a drug formulation of 2:1 weight ratio of FTC to glycerol excipient. The BIC-containing implants comprised 40 mm length, 200 µm wall thickness and a 5:1 weight ratio of BIC to castor oil excipient. The implant dimensions were selected based on prior studies and prescreening, where we have shown that implant geometry influences release behavior [9,12,13,14]. In general, longer implants provide greater drug reservoir capacity and release surface area, while thinner walls reduce the diffusion barrier and increase release rate. A 40 mm reservoir length was selected to provide sufficient load volume and the required release rate, and a wall thickness of 200–300 µm was chosen to balance release kinetics and structural integrity. Drug-to-excipient ratios were guided by prior formulation screening to balance drug dissolution within the reservoir and diffusion across the PCL membrane. Sufficient excipient was needed to wet the drug and maintain membrane-controlled, near zero-order release [13,14]. Prior to starting the in vitro studies, all the implants were packed within amber glass vials and sterilized by gamma irradiation (dose range 18–24 kGy) at Steris (Mentor, OH, USA) via continuous exposure to a Cobalt-60 gamma-ray source (Nordion Inc., Ottawa, ON, Canada) for 8 h.

In Vitro Drug Release Studies: The implants were placed in polypropylene tubes containing 1X phosphate-buffered saline (PBS) (pH 7.4) and incubated at 37 °C within an incubator shaker at 100 rpm. Although PBS does not fully simulate the subcutaneous interstitial fluid, it provides a standard and controlled in vitro dissolution medium to evaluate drug release under physiologic pH and sink conditions. The implants were transferred to fresh PBS buffer twice per week in a biosafety hood under aseptic conditions. The buffer volumes and transfer intervals were chosen to fully submerge implants and ensure sink conditions, as previously [13]. These studies used buffer volumes of 40 mL for FTC, and 200 mL for ISL and BIC. Sink conditions can be confirmed by calculating the minimum required volumes for a period of four days (the longest transfer interval) based on drug release rate and solubility, resulting in 0.152 mL for FTC, 7.36 mL for ISL, and 68.8 mL for BIC, all well below the buffer volumes used in these studies. The concentrations of ISL, BIC, and FTC in buffer release media were measured with an Agilent 1260 Infinity II HPLC-UV system (Agilent, Santa Clara, CA, USA) equipped with an Agilent Zorbax SB-C8 column (4.6 × 50, 3.5 µm). The mobile phases used were (A) 0.01% trifluoroacetic acid in water and (B) Acetonitrile. Separations were performed as follows: ISL (detection wavelength 260 nm, volume 20 µL, flow rate 0.8 mL/min, 86% MPA and 14% MPB); BIC (detection wavelength 320 nm, injection volume 30 µL, flow rate 0.8 mL/min, 40% MPA and 60% MPB); FTC (detection wavelength 285 nm, injection volume 50 µL, flow rate 1 mL/min, 97% MPA and 3% MPB).

In Vivo Absorption: IV bolus solutions were prepared as following: 1 mg/mL BIC solution in 2 mL of pure DMSO (Astatech, Bristol, PA, USA), 10 mg/mL ISL solution first prepared as a 30 mg/mL solution in DMSO and diluted 1:3 with normal saline (WuXi Apptec Co., Ltd., Wuhan, China), and 50 mg/mL FTC solution prepared in 2 mL of 0.9% PBS (BOC Sciences, Shirley, NY, USA). Twenty-four New Zealand White (NZW) rabbits were sourced from Charles River Laboratories Inc company for this study and treated according to the study schema (Supplementary Figure S1). Prior to IV bolus, the rabbits were sedated with 0.25–0.5 mg/kg acepromazine SQ, then placed in a rabbit restrainer. All ARV solutions were administered IV via the marginal vein of the ear with 8 rabbits per each of the following treatment groups: BIC 0.75 mg/kg only, ISL/FTC 5/30 mg/kg, and BIC/ISL/FTC 0.75/5/30 mg/kg. Two mL of whole blood was collected from the central auricular artery at the following timepoints, 30 min, and 1, 3, 6, 12, 18, 24, and 30 h using a sparse sampling design where each rabbit contributed 2 samples following a single IV bolus (total of 4 observations per treatment group per timepoint). These 24 rabbits subsequently underwent a washout period of 7 weeks and were then assigned to receive implants according to the following treatment groups (*n* = 8 per group): BIC only (via two BIC-eluting implants), ISL/FTC (via one ISL- and one FTC-eluting implant), and ISL/FTC/BIC (via one ISL-, one FTC- and one BIC-eluting implant). Six additional NZW rabbits assigned to the placebo group received implants containing excipients matched to those of the active treatment arms. The BIC, ISL, and FTC implants in each group contained an average mass of 165, 62, and 100 mg loaded, respectively. Each implant group contained equal numbers of males and females. The implants were placed in the SQ space between the shoulder blades using commercially available trocars. Bleeding occurred on days 1, 7, and 14, then every 14 days thereafter. Animals in the BIC-only group were terminated from the study at 56 days due to animal housing constraints. All other animals were followed for 1 year. Eight animals per dosing group were selected for the preclinical PK study to accommodate design aspects like sparse sampling and interim necropsy and based on expected variability in PK parameters along with common practice. Groups of 6–8 animals are frequently used to provide an adequate estimate of central tendency and variability while minimizing unnecessary animal use, consistent with ethical principles for animal research.

The rabbits were housed at the Tulane Vivarium according to AAALAC-accredited standards and monitored by veterinarians following IACUC-approved procedures and protocols (Protocol ID 923). The facility minimizes potential confounders through the strict control of environmental, feeding, and sample collection conditions. This research was conducted to comply with requirements set forth in the Animal Research: Reporting of In Vivo Experiments (ARRIVE) Guidelines. No a priori criteria were established for the inclusion or exclusion of animals and no formal randomization method was employed. Animal discomfort or pain was alleviated by the appropriate use of anesthesia or anesthetic medications, and all possible measures were taken to minimize discomfort for the animals. Adherence to IACUC protocols and SOPs was assessed by veterinary faculty and staff.

Bioanalytical Methods: BIC was extracted from rabbit plasma by protein precipitation with methanol containing the stable, isotopically labeled internal standard, BIC-d5. The samples were mixed and centrifuged prior to analysis by LC-MS/MS. Chromatographic separation was achieved by reverse phase chromatography on a Waters Atlantis T3, 2.1 × 50 mm, 3.0 µm analytical column with 0.1% formic acid in water (mobile phase A) and 0.1% formic acid in acetonitrile (mobile phase B) under gradient conditions injecting 4 µL. FTC and ISL were extracted from rabbit plasma by protein precipitation with methanol containing the stable, isotopically labeled internal standards, 13C15N2-FTC and 13C15N3-ISL. Following mixing and centrifugation, the extracts were evaporated to dryness, reconstituted with purified water, and transferred to a 96-well plate for LC-MS/MS analysis. Chromatographic separation was achieved by reverse phase chromatography on a Phenomenex Synergi Polar-RP, 2.0 × 50 mm, 2.5 µm analytical column with 0.1% acetic acid in water (mobile phase A) and methanol (mobile phase B) under gradient conditions injecting 10 µL. Detection of the analytes and internal standards were achieved on an AB Sciex API-5000 triple quadruple mass spectrometer under positive ion electrospray conditions. The transitions (*m*/*z*) monitored were as follows: BIC (450.2/289), BIC-d5 (455.2/294), ISL (294/154.1), 13C15N3-ISL (298/158), FTC (248/113), and 13C15N2-FTC (251/116). The calibration range for this assay was 1.00–1000 ng/mL (BIC) and 0.100–100 ng/mL (ISL and FTC). The overall intra-assay precision and accuracy results met 15% (20% LLOQ) acceptance criteria.

PK Modeling: All PK analyses were performed using PHOENIX WinNonlin/NLME^®^ version 8.3 (Certara Inc., Princeton, NJ, USA). Noncompartmental analysis (NCA) was performed for SQ and IV bolus plasma PK profiles, and values below the limit of quantification (BLQ) were imputed as half the lower limit of quantification (LLOQ) of the assay. Terminal elimination rate constants were estimated by fitting linear regression models to the terminal phase of the concentration vs. time profile on a natural log scale and were compared between routes of administration to confirm that the implant exhibited flip-flop kinetics where absorption was slower than elimination during subcutaneous administration. The IV bolus NCA parameter estimates were used as initial estimate to fit 1, 2, and 3-compartment macro-parameterized population PK models. The best fitting models were then used to estimate the mean unit impulse response (UIR) for all ARVs. Five rabbits (treated with ISL and FTC) were omitted from inclusion in the FTC IV PK model because all observations were BLQ for these animals. The models were evaluated based on parameter precision (relative standard error), goodness-of-fit diagnostics, and assessment of bias, with bootstrap analysis (250 resampled datasets with replacement) conducted as a supportive assessment of parameter stability.

Numerical deconvolution was performed using the deconvolution toolkit included in the PHOENIX WinNonlin/NLME software package version 8.3 with biexponential UIR functions for each ARV to estimate their respective SQ absorption profiles over time from their SQ concentration vs. time profiles. The UIR function was defined from the prospective PK model macro-parameters as the exponents (i.e., slope terms) and dose normalized coefficients. The latter are the y-intercepts associated with each exponential phase of the IV bolus concentration vs. time profile (i.e., A and B) divided by the administered IV bolus dose in ng/mL/ng units. We estimated absorption rate, cumulative mass absorbed, and fraction input every 72 h from day 1 up to day 371 based on the duration that each animal was observed. Deconvolution was not performed on animals with duplicative implants (i.e., those included in the BIC-only arm). These data were summarized by ARV as median and interquartile range.

Regression Analyses: Using the median results of the numerical deconvolution analysis from the subset of animals that were followed throughout the year-long study, we fit 6 dissolution models to characterize implant absorption kinetics using the least squares regression modeling package within the PHOENIX WinNonlin/NLME software package version 8.3. Of these dissolution models, we performed simple linear regression for zero-order, first-order, and Higuchi in the following forms; *F = b + mt*, *Ln*(*F_res_*) *= b + mt*, and *F = b + mt*^0.5^, respectively, where *F* is the fraction of the implant dose absorbed, *F_res_* is the fraction of the implant dose remaining, *b* is the *y*-axis intercept, *m* is the slope of the regression line, and *t* is time. Of the remaining dissolution models, we performed non-linear regression for Weibull, Double-Weibull, and Makoid–Banakar in the forms defined for Model 602, 603, and 604, respectively, by the Phoenix user guide [15] where *F* was set as the dependent variable and *t* as the independent variable. To characterize the relationship between in vivo absorption and in vitro dissolution, we also used this subset of animal’s mean fraction absorbed values estimated at timepoints corresponding to the in vitro sampling schedule of our parallel in vitro implant study. For this analysis, we fit 5 regression models (Supplementary Table S2) selected a priori based on the visual inspection of xy plots for fraction dissolved vs. fraction absorbed. The models were fit using the regression wizard tool within the SigmaPlot Version 16 Build 16.0.0.28 software package. For all regression analyses, the model selection criteria to determine the best fitting model were the correlation coefficient (r^2^) and Akaike Information Criterion (AIC).

Human Dose Translation: The SQ absorption rates estimated at 1, 3, 6, and 12 months post implantation were used to predict clinical plasma concentrations based on published pediatric PK parameter estimates or adult estimates when the former were not available. For these predictions, implant PK was assumed to follow a continuous, constant-rate infusion model according to $C_{ss}=\frac{k_{0}}{Cl}$ where *k*_0_ is the estimated absorption rate from deconvolution, *Cl* is the published apparent clearance estimate scaled for weight or bioavailability when necessary (Table 1), and *C_ss_* is the predicted pediatric steady-state plasma concentration. When required for scaling purposes weight was assumed as 15 kg for the target population based on CDC growth charts [16].

## 3. Results

**Figure 2:**
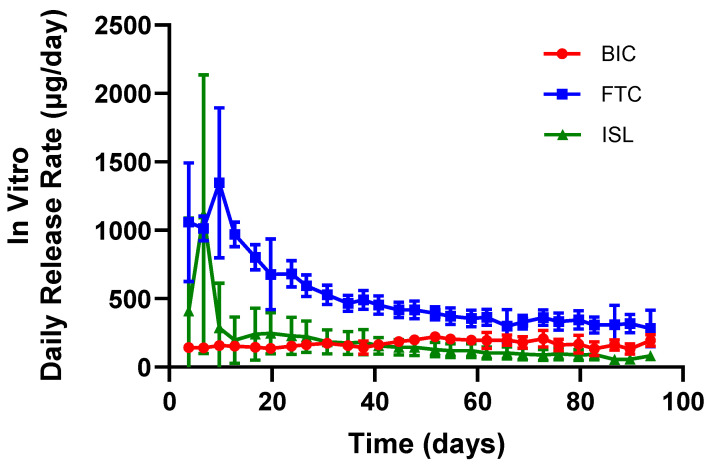
Mean (standard deviation) in vitro daily release rates of ARVs from implants (*n* = 7 ISL, *n* = 8 BIC and *n* = 8 FTC). Three implant designs, each containing a single ARV (ISL, FTC, or BIC), were tested under simulated physiological conditions.

In Vitro Dissolution from Implants: Implant parameters were initially selected based on published data [13] to achieve or exceed target daily inputs derived from pediatric oral PK. Three distinct implant designs were developed, with each implant containing a formulation of a single ARV (i.e., ISL, FTC, or BIC). The average daily in vitro dissolution rates are represented over time in Figure 2. The in vitro dissolution rate for ISL, FTC, and BIC calculated from day 14 to 90 correspond to 137 ± 54 µg/day, 430 ± 56 µg/day, and 172 ± 25 µg/day, respectively.

**Figure 3:**
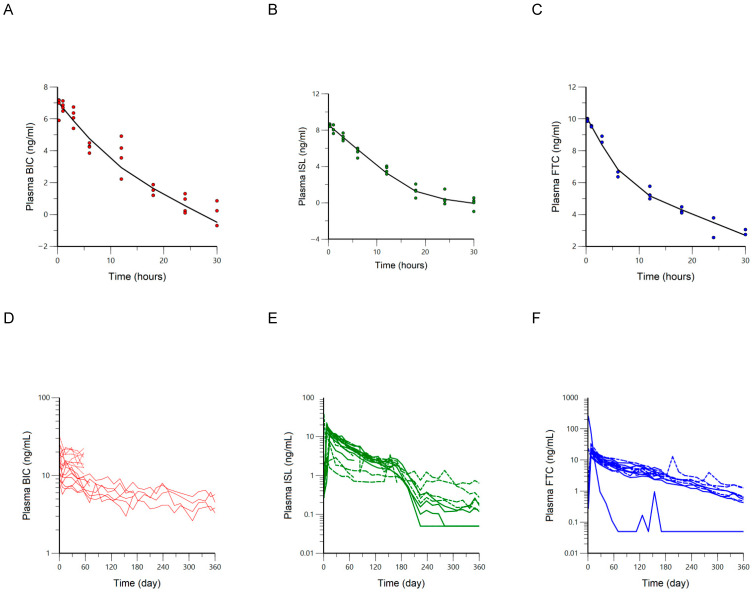
Plasma ARV concentrations are plotted on a natural log (Ln) scale over time following a single IV bolus dose (panels (**A**–**C**)) and on a log10 scale over time following SQ implantation (panels (**D**,**E**)). The solid black line in panels (**A**–**C**) represents the mean response for the sparsely sampled IV bolus data. Each individual animal’s full PK profile following SQ implantation is represented in panels (**D**–**F**) with animals in the ISL/FTC/BIC group represented by solid lines and those in the BIC only or ISL/FTC groups represented by dashed lines. Values below the limit of quantification were imputed as ½ LLOQ.

Plasma PK Profiles of IV Bolus and SQ Implants: The plasma drug concentrations for BIC, ISL, and FTC are plotted against time following IV bolus dosing and SQ implant administration (Figure 3). The plasma concentrations of the ARVs were above the limits of quantification for 30 h following IV bolus. The terminal elimination rate constant (λ_z_) estimates were significantly slower for the SQ route of administration compared to IV dosing (Table 2). This suggests that ARV dosing from the implant results in a flip-flop kinetic state, wherein the terminal phase of the concentration vs. time profile represents the rate-limiting step of absorption instead of elimination. The PK parameters estimated from the NCA were then used as initial parameter estimates for the compartmental PK models, whereupon the best model was selected to characterize drug disposition and generate the UIR required for deconvolution analysis. A two-compartment, macro-parameter population pharmacokinetic (popPK) model with log-additive error was used to fit the IV data of each ARV. BIC and ISL included random effects on all four parameters while a naïve-pooled approach was necessary to fit the FTC data. The diagnostic plots of the final models demonstrated adequate fit and minimal bias (Supplementary Figures S2 and S3). Most mean model parameter estimates fell within the 95% confidence intervals estimated from the bootstrap analyses (Table 3).

**Figure 4:**
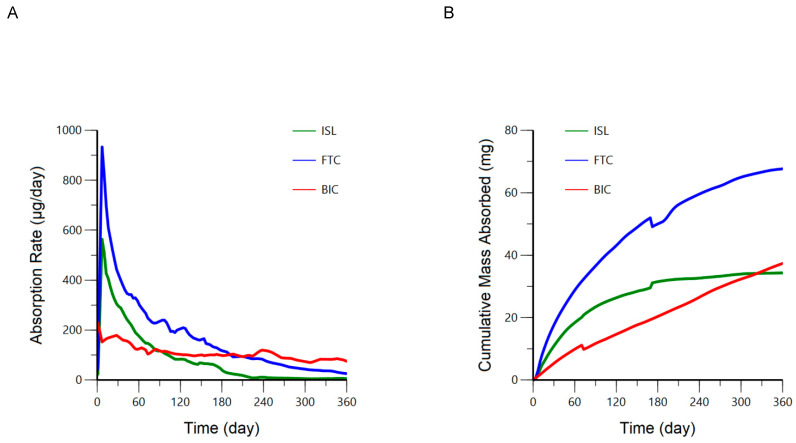
Median (IQR) absorption rates (**A**) and cumulative mass absorbed (**B**) following SQ administration of BIC-, ISL-, and FTC-eluting implants over 12 months. Red represents bictegravir (BIC; *n* = 8), green represents islatravir (ISL; *n* = 16), and blue represents emtricitabine (FTC, *n* = 16).

**Figure 5:**
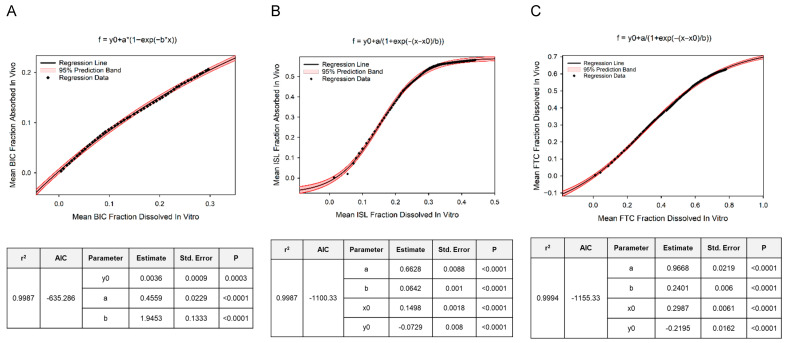
Best fitting dissolution vs. absorption model depicting the mean fraction absorbed in vivo as the dependent variable vs. fraction dissolved in vitro as the independent variable are plotted on a point-by-point basis for BIC (panel (**A**)), ISL (panel (**B**)), and FTC (panel (**C**)) with a solid black line representing the average regression line and the red shaded region representing the 95% prediction bands of the regression. The graph title indicates the best fitting regression curve, and parameter estimates along with fit statistics are tabulated below each graph for the respective ARV.

In Vivo Absorption Profiles of SQ Implants: Goodness-of-fit plots for the numerical deconvolution model demonstrated reasonable fit between individual predicted vs. observed plasma ARV concentrations over time following SQ administration (Supplementary Figures S3–S5). The median SQ absorption rates and cumulative mass absorbed over 12 months of implantation are represented graphically for BIC, ISL, and FTC in Figure 4 and as a snapshot analysis at 1, 3, 6, and 12 months in Table 4. Peak absorption rates were observed on day 1 for BIC and day 7 for ISL and FTC. The timeframe associated with the attainment of these peak absorption rates was defined as our “burst phase” and is reported accordingly in Table 4. Based on the cumulative mass absorbed, the respective percentage of the original ARV mass loaded into the implants remaining at 6 and 12 months is anticipated to be 81 and 65% for BIC, 48 and 45% for ISL, and 49 and 36% for FTC. Additionally, based on these estimated absorption rates, we anticipate BIC, ISL, and FTC plasma concentrations would match or exceed 24, 0.14, and 0.7 ng/mL for at least 6 months post implantation in our target population (Table 4). While a simple zero-order model fit BIC fraction absorbed vs. time data reasonably well (r^2^ = 0.9988, AIC = −709; Supplemental Table S1), our inspection of the residuals revealed non-linearity at the extremes of time, and the Weibull model exhibited the strongest fit (r^2^ = 0.9999, AIC = −982). The absorption kinetics of FTC and ISL were best characterized by the Double Weibull model (r^2^ = 0.9997, AIC = −747 and r^2^ = 0.9999, AIC = −892). We observed a strong and predictable relationship (albeit non-linear) between the mean in vitro fraction dissolved and the mean in vivo fraction absorbed for all three ARVs (Figure 5 and Supplemental Table S2). The three-parameter exponential rise to maximum model best described this relationship for BIC (adjusted r^2^ = 0.9987) while the four-parameter sigmoid model best described this relationship for ISL and FTC (adjusted r^2^ = 0.9987 and 0.9994, respectively).

## 4. Discussion

While approved pediatric HIV treatment options are generally safe and effective, the limited range of formulations and dosing regimens approved for this population pose challenges. Palatability and ease of administration are unique determinants of adherence in pediatric populations and are associated with suboptimal treatment outcomes [2]. Although LA HIV treatment regimens, such as the injectable Cabenuva, could address these challenges, no such formulations have been approved for pediatric use to date. To this end, we have developed the LA drug-delivery platform to provide safe and effective HIV treatment through a subcutaneously placed reservoir-style implant for children.

This preclinical work establishes proof-of-concept that, with additional tuning of the delivery system, our platform could support the sustained release of a full HIV treatment regimen comprising an INSTI and two NRTIs while maintaining plasma concentrations within the therapeutic window for young children. We used a year-long study in NZW rabbits and PK modeling to fully characterize the ARV absorption profiles achieved by our implants, with daily SQ doses at 6 months post implantation estimated as 98, 37, and 116 μg/day of BIC, ISL, and FTC, respectively. We translated these SQ doses based on the available PK literature (Table 1) and predict that at 6 months post implantation these implants would achieve plasma concentrations in a pediatric population of 24, 0.14 and 0.7 ng/mL, for BIC, ISL and FTC, respectively. While these plasma concentrations are lower than the typical trough plasma concentrations achieved by daily oral dosing, they are likely within the range of C_min_ values yielded when a patient misses a dose. Importantly, oral dosing regimens tested in pediatric clinical trials are typically based on the effective doses identified in Phase II and III trials in adults, rather than a specifically established pediatric concentration–response relationship. Moreover, in drug development, oral doses are often designed to include PK buffer in case a patient misses a dose. One advantage of the implant system is that missed doses are not possible; therefore, implant dosing can be more fine-tuned to the concentration vs. response relationship. The predicted pediatric concentrations achieved by our implant system over 6 months fell within the range of monotherapy 50% efficacy concentration (EC_50_) values reported for wild-type HIV for BIC (protein-adjusted median, range = 11, 1–33 ng/mL [17,19]), ISL (range = 0.014–0.88 ng/mL [20]), and FTC (median, range = 2.3, 0.25–7.4 ng/mL [17]). Importantly, synergistic interactions between BIC, FTC and tenofovir alafenamide (TAF) have been previously published [19]. Because pharmacodynamic interactions between BIC, ISL and FTC may decrease the concentration requirements for efficacy, optimized implant dosing would target combination efficacy thresholds rather than monotherapy efficacy thresholds. This is important for the implant system because the daily dosing rate is inversely related to the duration of ARV delivery. Thus, this dose optimization effort is an opportunity to achieve a longer duration of effective ARV delivery. In lieu of this anticipated synergy between these ARVs, it is reasonable to expect the potency yielded by our implant to be higher than predicted by benchmarking against previously published monotherapy effective concentrations.

Following reports of decreased lymphocyte counts from clinical trials in participants taking oral ISL doses exceeding 20 mg per week or 60 mg per month for over 24 months [21], ISL’s clinical development as a pre-exposure prophylaxis (PrEP) agent was placed on a voluntary clinical hold by the manufacturer. Subsequently, PK modeling and a Phase 2 trial with 96 weeks of follow-up have demonstrated that oral ISL doses of 2 mg per week will be safe for adults [22]. While the safe oral dose of ISL has yet to be defined for a pediatric population, we are encouraged that the cumulative dose of ISL yielded by our implant over the 7-day “burst” period is approximately 10 times lower than the oral dosing level associated with lymphocytopenia after 24 repeated dosing events.

There are some notable findings from this analysis to support our approach for the development of this distinctive technology. First, we confirm that our implant technology results in a flip-flop kinetic state wherein the implant’s controlled-release mechanism yields a rate-limiting absorption profile that sustains drug exposure in the body, allowing for longer dosing intervals. Second, we used compartmental PK modeling to identify UIR functions that can support the numerical deconvolution of BIC, ISL and FTC absorption rates within the rabbit model and facilitate dose translation to our target pediatric population. Third, we harnessed this deconvolution model to fully characterize absorption kinetics as well as the point-to-point relationship between in vitro dissolution and in vivo absorption for our implant formulations.

Previous in vivo characterization conducted by Li et al. demonstrated long-acting release over 6 months of co-formulated levonogestrel, etonogestrel, and ISL from a similar implant in female Wistar rats [14]. Our study contained differences in ISL formulation and implant wall dimensions which may have contributed to an estimated faster absorption rate initially, though the cumulative drug released around 1 year is approximately 30 mg for both studies. Our findings align with this previous report in that: the quantification of the implant ARVs in plasma was sustained and, here, extended beyond one year. Notably the implant described here and that described by Li et al. exhibited a similar cumulative drug released around 1 year (~30 mg) despite the current implant being loaded with less ISL (mean 62 mg vs. 110 mg). The tunability of the drug’s transport out of implant is influenced by multiple factors (polymer composition, drug physiochemical properties, implant surface area and thickness, and the ratio of excipient to drug). The unique absorption rate profiles for each ARV in Figure 4 are likely driven by multiple factors, including the distinct physiochemical properties of each drug [23].

There are some limitations to this study. First, the IV model was developed from sparse preclinical data (16 rabbits per ARV group, each contributing one PK sample at two timepoints). This study schema was implemented to align with respective animal safety protocols. Despite this, parameter estimates were considered reliable based on (a) acceptable precision (most parameters had CV% < 40%) and (b) the absence of systematic biases in model diagnostics. When there was insufficient data to build a popPK model (e.g., FTC), we used a naïve-pooled approach (no interindividual variability) to model the IV data. This occurred because five rabbits had a high prevalence of BLQ values in unexpected portions of the concentration–time curve, which we believe to reflect unreliable dosing information. Model evaluation emphasized goodness-of-fit, parameter precision (RSE%), and lack of bias. Bootstrap resampling was therefore used as a supportive assessment of parameter stability as the primary objective was to support a proof-of-concept deconvolution framework rather than formal inference on parameter uncertainty. Consistency between the bootstrap and final parameter estimates supports that the model is fit for purpose. The bootstrap size (*n* = 250) reflects a pragmatic balance given the exploratory nature of the analysis and data limitations. Second, deconvolution estimates drug absorption by relying on assumptions that are not required in traditional mass-balance assessments. Deconvolution assumes (a) linear drug distribution and elimination (PK at different doses is consistent), (b) drug molecule movement is independent of one another and only depends on time since administration, (c) probabilistic drug transport based on stochastic approximations instead of exact mechanisms. Furthermore, deconvolution has not historically been used in implant research. However, given that the main requirement for deconvolution is a representative UIR function (most commonly derived through a single IV bolus administration) and that the ARVs tested herein are not known to auto-induce or -inhibit their own metabolism, we believe this approach can be applied for longer-acting drug formulations such as ours [24]. Additionally, the visual inspection of residual plots for our best fitting non-linear regression models to describe mean fraction absorbed vs. dissolved revealed some patterns of variance suggesting room for parameter refinement. However, given our inability to assess whether this relationship holds true across multiple formulations and species, we believe the parameters reported here are fit-for-purpose as a first step towards characterizing our IVIVC. These parameters will provide adequate prior knowledge to help narrow our IVIVC model search as more data become available. Lastly, although the pharmacodynamic activity of ISL and FTC is directly related to their active intracellular phosphorylated metabolites, we did not measure these and instead use plasma concentrations of the parent compounds as surrogates. Our previous studies have demonstrated inter-species differences in the phosphorylation of ISL with rabbits having the lowest capacity to phosphorylate this compound among the animal models tested [25]. Thus, the metabolite data yielded within this study would be of low clinical relevance. Further, the assessment of the implant platform in animal models, such as nonhuman primates, that are more translatable to human pharmacology will be an important next step in predicting the likelihood of efficacious ARV delivery by this implant system to our target population.

## 5. Conclusions

Overall, this study shows how PK modeling can guide the design of new LA drug delivery systems. By establishing a modeling framework to support numerical deconvolution, it becomes possible to accurately estimate absorption profiles from non-IV and LA formulations without depending on the assumption of zero-order absorption as required by traditional, mass-balance estimation of absorption rates. For our technology, independence of this assumption is particularly important given that the ARV absorption kinetics achieved by our implant were not reliably described by the zero-order model. Here, we translated these ARV absorption rates to predict ARV concentrations that can be achieved by our implant system within a target pediatric population. Such insights can inform and refine product development, ensuring implant specifications meet clinical dosing requirements. Finally, we developed mathematical models to facilitate the prediction of the vivo absorption profile via the in vitro dissolution profile. While additional studies are needed to fully validate this IVIVC for regulatory purposes, our findings represent an important first step towards the development of a comprehensive in silico NAM to bypass downstream animal studies as we advance our technology towards clinical trials. This integrated approach also aligns with future regulatory expectations to facilitate dose scaling, and rationale optimization of LA-ART delivery products.

## Figures and Tables

**Table 1:** Approach to clinical translation of dose from preclinical data.

Published PK Data	BIC	FTC	ISL
Reference	BIKTARVY^®^ Prescribing Information [17]		Schurmann et al. [18]
Study Population	3- to 9-year-old children		18–60-year-old adults
Population Weight	14 to <25 kg		Not reported
Study Treatment	Biktarvy (BIC/TAF/FTC) FDC oral tablet		Islatravir oral capsules
Study Dose(s)	30 mg	120 mg	0.5, 1, 2, 10, and 30 mg
Published Parameter	AUC_24h_ = 126 μg * h/mL	AUC_24h_ = 15 μg * h/mL	CL/F = 35,860 mL/h ^a^
Assumed Bioavailability	70%	93%	100%
Pediatric Cl Calculation	$Cl=\frac{Dose*F}{AUC}$		${Cl}_{ped}=\;{Cl}_{adult}*(\frac{{Weight}_{pediatric}\;b}{{Weight}_{adult}\;c}$)^0.75^
Pediatric Cl Estimate	167 mL/h	7440 mL/h	11,294 mL/h

FDC = fixed dose combination, ^a^ = average of CL/F estimates reported for each dosing level, ^b^ Weight_pediatric_ assumed 15 kg, ^c^ Weight_adult_ assumed 70 kg.

**Table 2:** Comparison of terminal slopes calculated and imputed concentration count during NCA of BIC, ISL and FTC in rabbit after different dosing routes.

ARV	NCA Parameter	Route	
		SQ Implant *	IV Bolus **
BIC	λ_z_, 1/h	6.0 × 10^−4^	0.181
	No. (%) imputed BLQ values	0 (0%)	1 (3.1%)
ISL	λ_z_, 1/h	3.7 × 10^−4^	0.216
	No. (%) imputed BLQ values	17 (5.7%)	0 (0%)
FTC	λ_z_, 1/h	1.1 × 10^−4^	0.154
	No. (%) imputed BLQ values	18 (6%)	0 (0%)

* SQ estimates represent the median NCA values of the individual profiles (*n* = 16 rabbits) spanning 0 to >1 year. ** IV estimates represent the mean NCA value of the group profile (*n* = 16 rabbits) using the sparse sampling option and spanning 0–30 h. Five rabbits were omitted from inclusion in the emtricitabine pharmacokinetic model because all observations were BLQ for these animals.

**Table 3:** Final popPK macro-parameter estimates with bootstrap estimates for BIC, ISL, and FTC in rabbits.

ARV	Parameter	Final Estimate, %CV	Bootstrap Estimate	Bootstrap 95% CI
BIC	A (ng/mL)	1115.6 (39)	1178	(816–1581)
	Alpha (1/h)	0.438 (20)	0.430	(0.320–0.562)
	B (ng/mL)	102.0 (98)	79.5	(35.7–141.1)
	Beta (1/h)	0.170 (23)	0.159	(0.119–0.188)
ISL	A (ng/mL)	5275.9 (9)	5358	(4742–6162)
	Alpha (1/h)	0.5 (6)	0.45	(0.41–0.49)
	B (ng/mL)	6.3 (69)	7	(4–12)
	Beta (1/h)	0.1 (40)	0.06	(0.04–0.09)
FTC	A (ng/mL)	25,323.5 (6)	25,148	(22,491–28,776)
	Alpha (1/h)	0.6 (10)	0.64	(0.50–0.81)
	B (ng/mL)	810.5 (29)	859	(380–1625)
	Beta (1/h)	0.1 (12)	0.13	(0.10–0.18)

**Table 4:** Median (IQR) subcutaneous absorption rates over 12 months.

ARV	N *	Month	Absorption Rate (μg/Day)	Cumulative Mass (mg)	Predicted Pediatric C_ss_ (ng/mL)
BIC	8	Burst ^ŧ^	417.8 (364.0–524.2)	0.09 (0.07–0.11)	104.2
	8	1	172.5 (129.6–201.2)	5.5 (4.1–6.3)	43.0
	6	3	116.0 (97.9–139.1)	11.8 (10.6–16.1)	28.9
	4	6	97.6 (83.8–120.5)	20.5 (18.0–28.2)	24.4
	4	12	70.9 (58.2–95.6)	37.7 (31.8–46.9)	17.7
ISL	16	Burst ^ŧ^	563.2 (66.0–687.8)	1.77 (0.61–2.15)	2.08
	16	1	294.8 (160.4–339.3)	11.2 (6.7–12.6)	1.09
	12	3	116.0 (91.7–131.3)	23.5 (17.3–25.3)	0.43
	8	6	37.4 (28.1–50.3)	31.5 (25.3–33.9)	0.14
	8	12	4.7 (2.2–7.9)	34.3 (27.0–35.7)	0.02
FTC	16	Burst ^ŧ^	933.2 (804.7–1105.5)	3.0 (2.66–3.45)	5.2
	16	1	420.5 (353.3–503.5)	18.1 (16.0–21.2)	2.4
	12	3	236.2 (161.3–267.5)	36.8 (31.9–44.9)	1.3
	8	6	116.6 (85.3–147.4)	50.1 (45.9–62.3)	0.7
	8	12	24.1 (18.6–46.7)	67.8 (57.0–74.1)	0.1

* N = number of rabbits at respective timepoint, ^ŧ^ Burst was defined as the timepoint associated with the peak median absorption rate for each ARV. This was day 1 for BIC and day 7 for ISL and FTC.

## Data Availability

The original contributions presented in this study are included in the article/Supplementary Material. Further inquiries can be directed to the corresponding author.

## Supplementary Materials

The following supporting information can be downloaded at https://www.mdpi.com/article/10.3390/pharmaceutics18050522/s1, Figure S1: Schematic Diagram for IV Bolus and Subcutaneous Implant Studies in Rabbits; Figure S2: Bictegravir Model Diagnostics; Figure S3: Islatravir Model Diagnostics; Figure S4: Emtricitabine Model Diagnostics; Figure S5: Bictegravir Deconvolution Goodness of Fit; Figure S6: Islatravir Deconvolution Goodness of Fit; Figure S7: Emtricitabine Deconvolution Goodness of Fit; Table S1: Parameter Estimates and Diagnostics for Absorption Models; Table S2: Parameter Estimates and Diagnostics for Absorption vs. Dissolution Models.
