# Supplementary material for: Harnessing Pharmacokinetic Modeling to Develop a Long-Acting Subcutaneous HIV Treatment Platform for Young Children
Source: Pharmaceutics. 2026 Apr 24;18(5):522. doi: 10.3390/pharmaceutics18050522 (PMC13210555; doi:10.3390/pharmaceutics18050522)
Supplement: Supplementary file 1 [file pharmaceutics-18-00522-s001.zip › pharmaceutics-4189523-supplementary.pdf]

SUPPLEMENTARY DATA

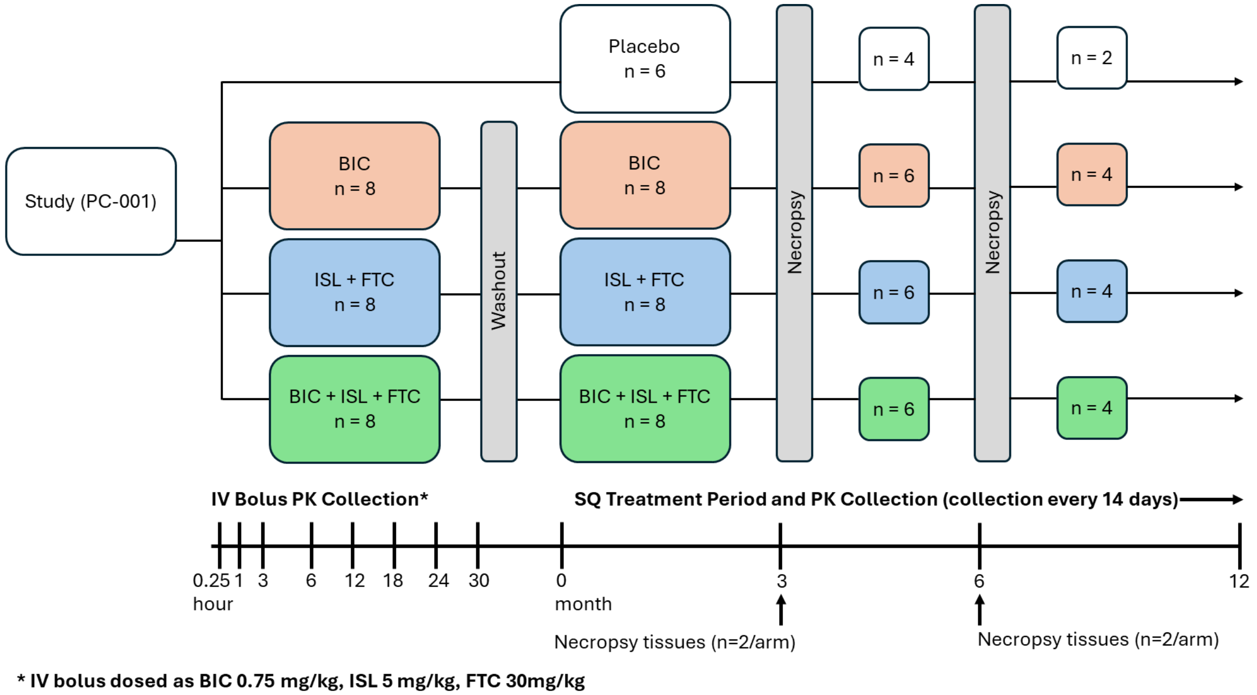

Figure S1: Schematic Diagram for IV Bolus and Subcutaneous Implant Studies in Rabbits

SUPPLEMENTARY FIGURE S1. In-vivo study design schematic showing groups of NZW rabbits receiving 1 of 3 treatments alongside a placebo group (excipient only). Two animals were randomly selected within each treatment group for two scheduled necropsies at month 3 and 6. BIC = bictegravir, ISL = islatravir, FTC = emtricitabine, IV = intravenous, n = number of rabbits, SQ = subcutaneous

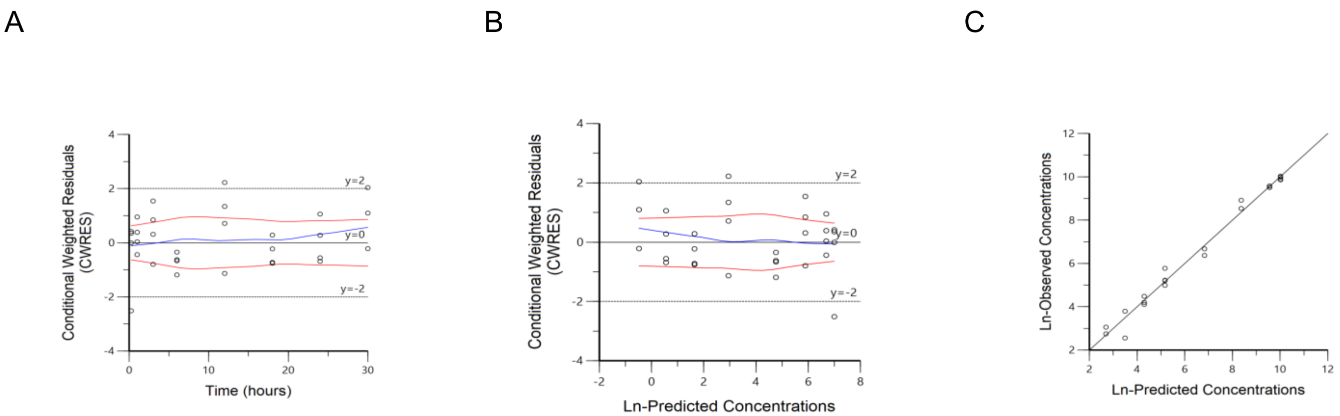

SUPPLEMENTARY FIGURE S2. Bictegravir model diagnostics. Panel A is conditional weighted residuals vs time with loess fit (blue) and absolute trend lines (red). Panel B is weighted residuals vs ln-transformed predicted concentrations with loess fit (blue) and absolute trend lines (red). Panel C is ln-transformed observed vs predicted concentrations (open circles) with the ln- prediction curve (black line).

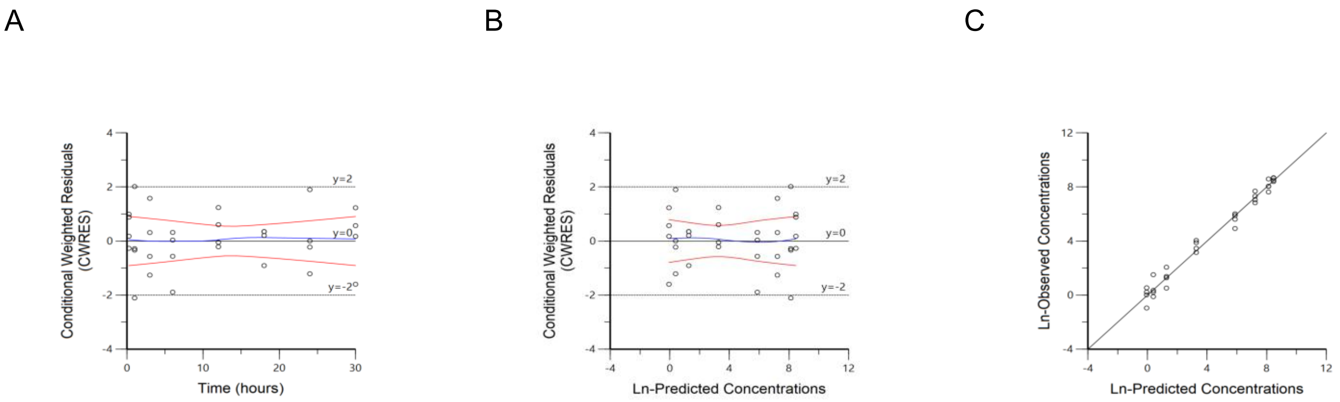

SUPPLEMENTARY FIGURE S3. Islatravir model diagnostics. Panel A is conditional weighted residuals vs time with loess fit (blue) and absolute trend lines (red). Panel B is weighted residuals vs Ln-transformed predicted concentrations with loess fit (blue) and absolute trend lines (red). Panel C is Ln-transformed observed vs predicted concentrations (open circles) with the ln- prediction curve (black line).

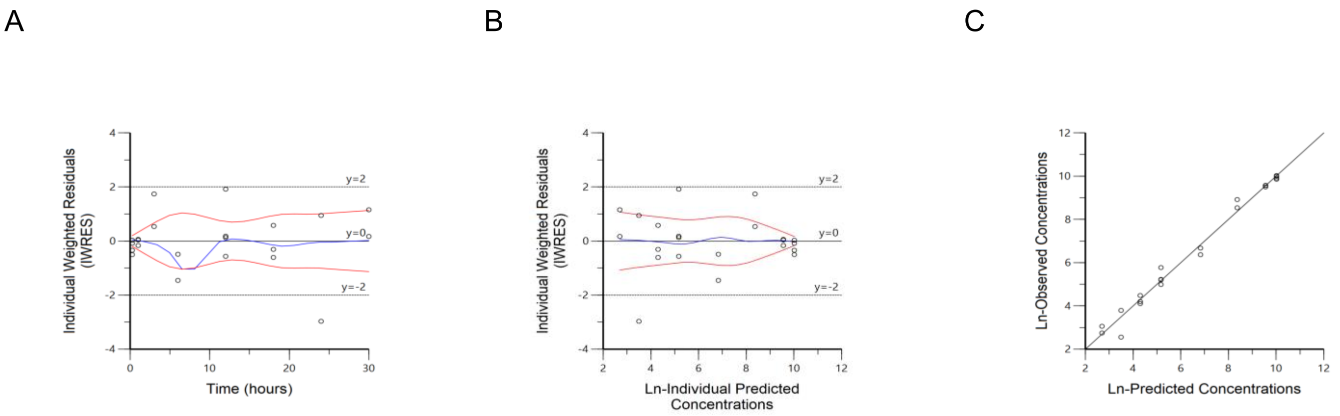

SUPPLEMENTARY FIGURE S4. Emtricitabine model diagnostics. Panel A is conditional weighted residuals vs time with loess fit (blue) and absolute trend lines (red). Panel B is weighted residuals vs Ln-transformed predicted concentrations with loess fit (blue) and absolute trend lines (red). Panel C is Ln-transformed observed vs predicted concentrations (open circles) with the ln- prediction curve (black line).

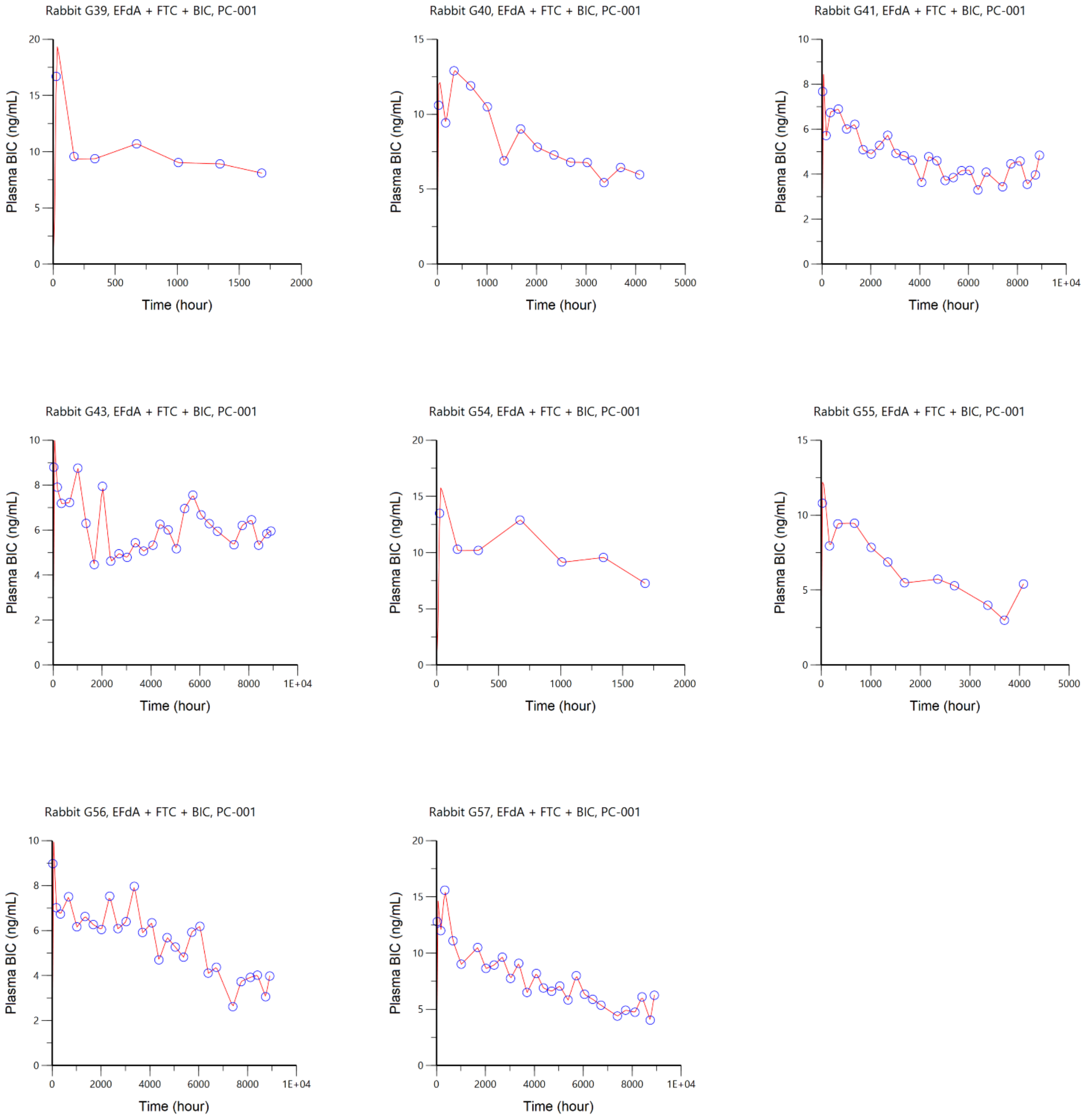

SUPPLEMENTARY FIGURE S5. Bictegravir Deconvolution Goodness of Fit. Observed time-concentration data (blue circles) vs. the predicted curve (red line) for bictegravir in rabbits (n = 8)

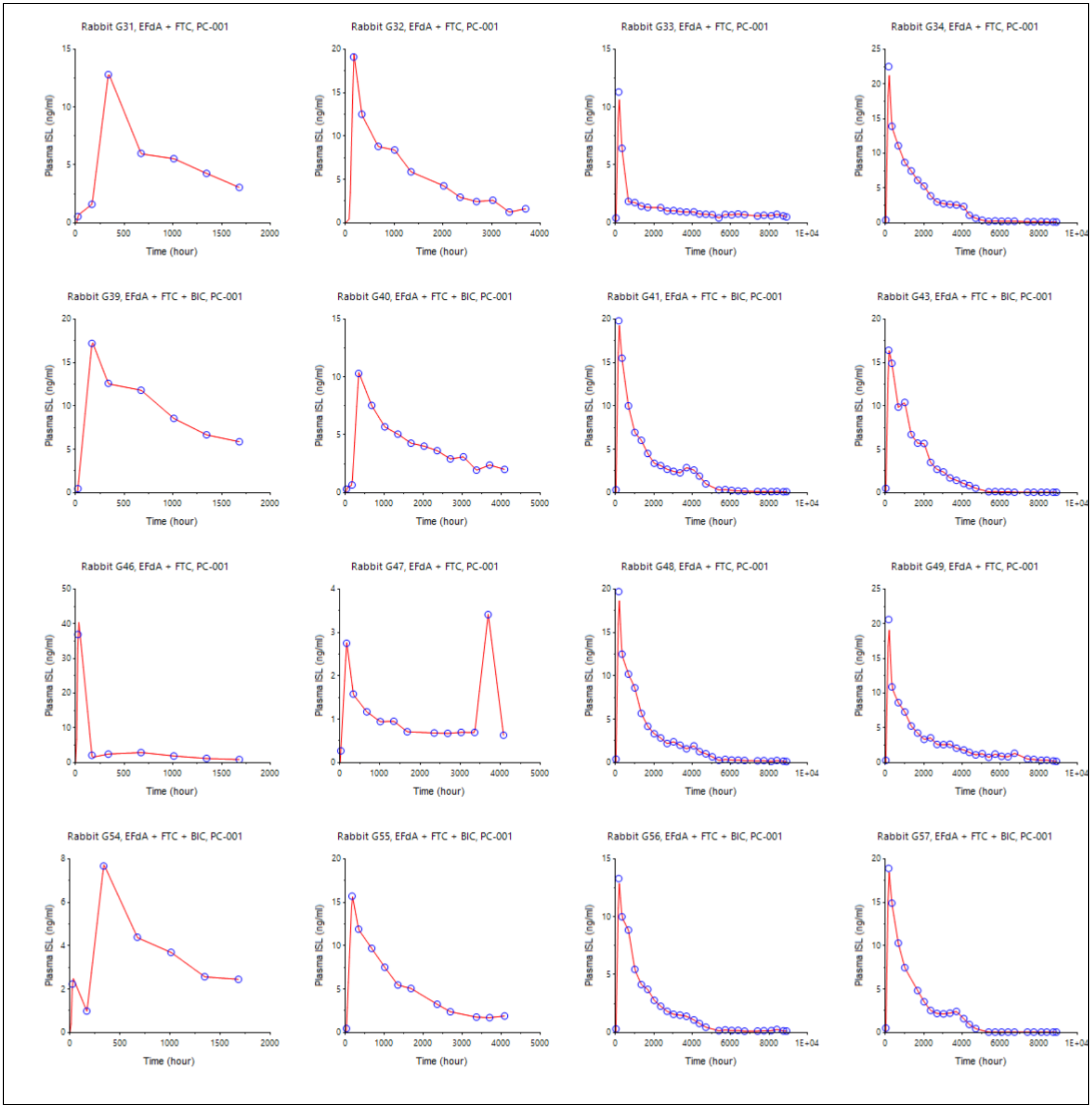

SUPPLEMENTARY FIGURE S6. Islatravir Deconvolution Goodness of Fit. Observed time-concentration data (blue circles) vs. the predicted curve (red line) for islatravir in rabbits (n = 16)

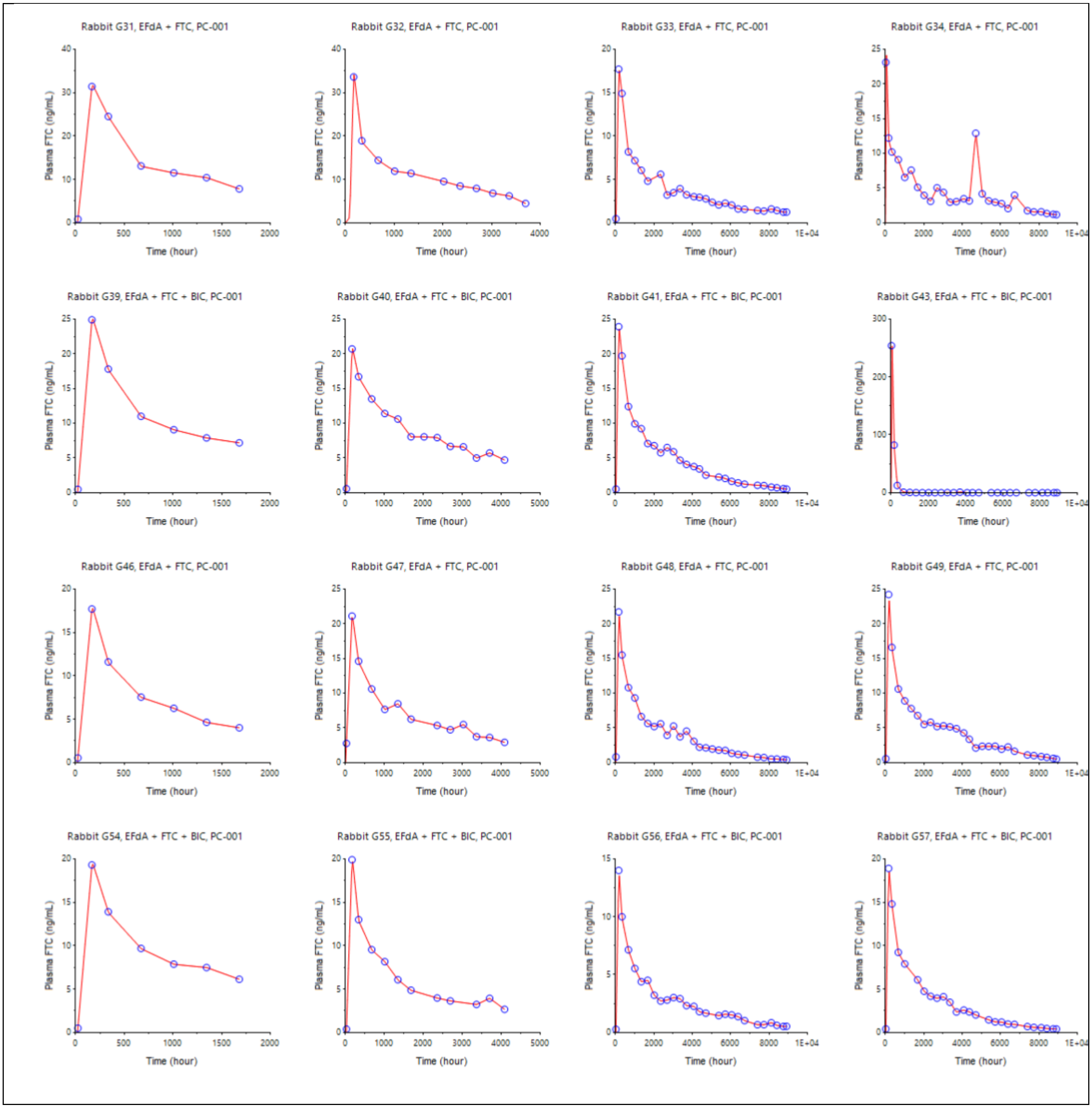

SUPPLEMENTARY FIGURE S7. Emtricitabine Deconvolution Goodness of Fit. Observed time-concentration data (blue circles) vs. the predicted curve (red line) for emtricitabine in rabbits (n = 16)

Supplementary Table S1 Parameter Estimates and Diagnostics for Absorption Models

ARV	Model	r^2	AIC	Parameter	Estimate	Std. Error
BIC	Zero-Order	0.9988	-709	INT	0.014134	0.000915
				SLOPE	3.94E-05	1.78E-07
	First Order	0.9693	-274	INT	-0.00064	0.000435
				SLOPE	-4.89E-05	8.46E-08
	Higuchi	0.9872	-419	INT	-0.093	0.004395
				SLOPE	0.004497	6.59E-05
	**Weibull**	**0.9999**	**-981**	**FINF**	**1.666046**	**0.178462**
				**MDT**	**39994.39**	**5537.618**
				**B**	**0.949174**	**0.007761**
				**TLAG**	**0**	
				**INT**	**0**	
	Double Weibull	0.9465	-187	F1	0.500032	2.50E+10
				FINF	0.285384	0.158092
				MDT1	2840.739	1.74E+11
				B1	1.033039	19019693
				MDT2	2840.737	1.74E+11
				B2	1.033038	19059146
				TLAG	0	
				INT	0	
	Makaoid-Banakar	0.9997	-865	FMAX	0.784593	0.055427
				TMAX	45517.66	4593.658
				B	0.968058	0.012035
				TLAG	0	
				INT	0	
FTC	Zero-Order	0.9352	-105	INT	0.187129	0.010423
				SLOPE	5.91E-05	2.03E-06
	First Order	0.9693	-274	INT	1.921903	0.005278
				SLOPE	-4.46E-05	1.03E-06
	Higuchi	0.986	-292	INT	-0.00161	0.007336
				SLOPE	0.007186	0.00011
	Weibull	0.999	-618	FINF	0.675992	0.005262
				MDT	3101.332	56.46712
				B	0.89279	0.01082
				TLAG	0	
				INT	0	
	**Double Weibull**	**0.9997**	**-746**	**F1**	**0.432891**	**0.047613**
				**FINF**	**0.613557**	**0.001919**
				**MDT1**	**4616.781**	**162.8111**
				**B1**	**2.397683**	**0.197402**
				**MDT2**	**1095.531**	**112.6986**
				**B2**	**1.127737**	**0.036545**
				**TLAG**	**0**	
				**INT**	**0**	
	Makaoid-Banakar	0.9992	-634	FMAX	0.619497	0.002416
				TMAX	9997.339	158.6251
				B	0.783245	0.009056
				TLAG	0	
				INT	0	
ISL	Zero-Order	0.8311	-40	INT	0.254818	0.013589
				SLOPE	4.36E-05	2.64E-06
	First Order	0.8746	-164	INT	1.868785	0.008214
				SLOPE	-3.18E-05	1.60E-06
	Higuchi	0.9253	-134	INT	0.096892	0.01387

				SLOPE	0.0056	0.000208
	Weibull	0.9995	-751	FINF	0.547635	0.000806
				MDT	1612.668	7.686005
				B	1.024965	0.006938
				TLAG	0	
				INT	0	
	**Double Weibull**	**0.9999**	**-891**	**F1**	**0.656434**	**0.040172**
				**FINF**	**0.544194**	**0.000568**
				**MDT1**	**2288.246**	**95.21243**
				**B1**	**1.426693**	**0.066116**
				**MDT2**	**589.4632**	**28.99826**
				**B2**	**1.400658**	**0.04603**
				**TLAG**	**0**	
				**INT**	**0**	
	Makaoid-Banakar	0.9986	-615	FMAX	0.538638	0.001015
				TMAX	5843.568	72.96909
				B	0.824929	0.01201
				TLAG	0	
				INT	0	

Supplementary Table S2 Parameter Estimates and Diagnostics for Absorption vs Dissolution Models

ARV	Model	Equation	$AdjR^2$	AIC	Parm	Estimate	StdError	P value
BIC	Linear	f = y0+a*x	0.9936	-551	y0	0.0138	0.0013	<0.0001
					a	0.6683	0.0075	<0.0001
	3-parameter Sigmoid	f = a/(1+exp(-(x-x0)/b))	0.9842	-503	a	0.2153	0.0063	<0.0001
					b	0.0674	0.0035	<0.0001
					x0	0.1377	0.0054	<0.0001
	4-parameter Sigmoid	f = y0+a/(1+exp(-(x-x0)/b))	0.9987	-631	a	8.3113	118.883	0.9446
					b	0.4737	0.5671	0.4077
					x0	-1.3637	8.5549	0.874
					y0	-7.8654	118.6791	0.9474
	2-parameter Exponential	f = a*(1-exp(-b*x))	0.9984	-624	a	0.4046	0.0131	<0.0001
					b	2.3385	0.0986	<0.0001
	3-parameter Exponential	f = y0+a*(1-exp(-b*x))	0.9987	-635	y0	0.0036	0.0009	0.0003
					a	0.4559	0.0229	<0.0001
					b	1.9453	0.1333	<0.0001
FTC	Linear	f = y0+a*x	0.9878	-838	y0	0.0185	0.005	0.0003
					a	0.8329	0.0091	<0.0001
	3-parameter Sigmoid	f = a/(1+exp(-(x-x0)/b))	0.9958	-948	a	0.673	0.0053	<0.0001
					b	0.1587	0.003	<0.0001
					x0	0.379	0.0034	<0.0001
	4-parameter Sigmoid	f = y0+a/(1+exp(-(x-x0)/b))	0.9994	-1155	a	0.9668	0.0219	<0.0001
					b	0.2401	0.006	<0.0001
					x0	0.2987	0.0061	<0.0001
					y0	-0.2195	0.0162	<0.0001
	2-parameter Exponential	f = a*(1-exp(-b*x))	0.9936	-905	a	2.1612	0.1806	<0.0001
					b	0.4603	0.0445	<0.0001
	3-parameter Exponential	f = y0+a*(1-exp(-b*x))	0.9966	-971	y0	-0.0475	0.0052	<0.0001
					a	1.4026	0.0558	<0.0001
					b	0.8741	0.0544	<0.0001
ISL	Linear	f = y0+a*x	0.8687	-620	y0	0.0977	0.0152	<0.0001
					a	1.2846	0.0489	<0.0001
	3-parameter Sigmoid	f = a/(1+exp(-(x-x0)/b))	0.9971	-1021	a	0.5836	0.0015	<0.0001
					b	0.0561	0.0007	<0.0001
					x0	0.1654	0.0007	<0.0001
	4-parameter Sigmoid	f = y0+a/(1+exp(-(x-x0)/b))	0.9987	-1100	a	0.6628	0.0088	<0.0001
					b	0.0642	0.001	<0.0001
					x0	0.1498	0.0018	<0.0001
					y0	-0.0729	0.008	<0.0001
	2-parameter Exponential	f = a*(1-exp(-b*x))	0.9439	-709	a	0.848	0.0399	<0.0001
					b	2.9924	0.238	<0.0001
	3-parameter Exponential	f = y0+a*(1-exp(-b*x))	0.9741	-789	y0	-0.1829	0.0189	<0.0001
					a	0.8803	0.0142	<0.0001
					b	5.1965	0.267	<0.0001
